# Crystal structure of methyl (2*Z*)-2-[(2*Z*)-2-(2-cyclo­pentyl­idenehydrazin-1-yl­idene)-4-oxo-3-phenyl-1,3-thia­zolidin-5-yl­idene]ethano­ate

**DOI:** 10.1107/S2056989015017454

**Published:** 2015-09-26

**Authors:** Mehmet Akkurt, Victoria A. Smolenski, Shaaban K. Mohamed, Jerry P. Jasinski, Alaa A. Hassan, Mustafa R. Albayati

**Affiliations:** a Department of Physics, Faculty of Sciences, Erciyes University, 38039 Kayseri, Turkey; b Department of Chemistry, Keene State College, 229 Main Street, Keene, NH 03435-2001, USA; c Chemistry and Environmental Division, Manchester Metropolitan University, Manchester M1 5GD, England; d Chemistry Department, Faculty of Science, Minia University, 61519 El-Minia, Egypt; e Kirkuk University, College of Science, Department of Chemistry, Kirkuk, Iraq

**Keywords:** crystal structure, thia­zolidinyl ring, disorder, hydrogen bonding, C—H⋯π inter­actions

## Abstract

In the title compound, C_17_H_17_N_3_O_3_S, the cyclo­pentane ring is disordered over two sets of sites with an occupancy ratio of 0.775 (8):0.225 (8) for the affected atoms. The thia­zolidinyl ring is planar (r.m.s. deviation = 0.024 Å) and forms a dihedral angle of 65.13 (8)° with the attached phenyl ring. The mol­ecular packing is stabilized by C—H⋯O and C—H⋯π inter­actions, forming a three-dimensional structure.

## Related literature   

For biological properties of thia­zole-containing compounds, see: Quiroga *et al.* (2002 ▸); Hutchinson *et al.* (2002 ▸); Hargrave *et al.* (1983 ▸); Patt *et al.* (1992 ▸); Sharma *et al.* (2009 ▸); Jaen *et al.* (1990 ▸); Tsuji & Ishikawa (1994 ▸); Bell *et al.* (1995 ▸): Ergenc *et al.* (1999 ▸); Carter *et al.* (1999 ▸); Badorc *et al.* (1997 ▸); Rudolph *et al.* (2001 ▸).
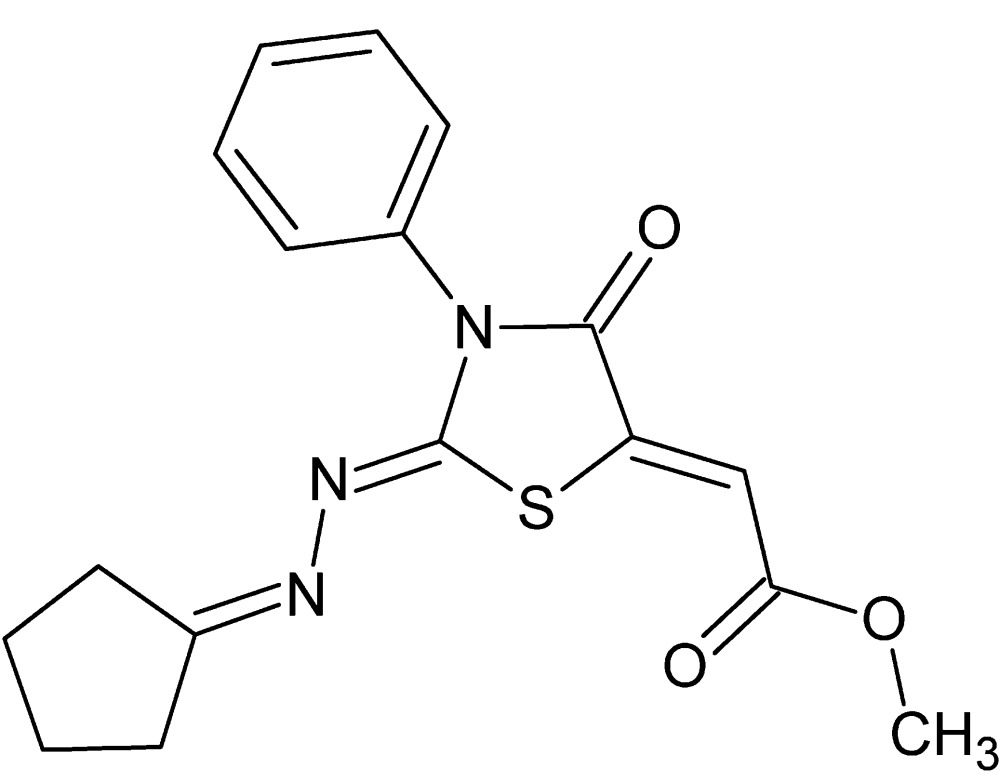


## Experimental   

### Crystal data  

C_17_H_17_N_3_O_3_S
*M* *_r_* = 343.40
Monoclinic, 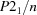

*a* = 5.5215 (3) Å
*b* = 16.1299 (8) Å
*c* = 18.7112 (9) Å
β = 93.980 (5)°
*V* = 1662.42 (15) Å^3^
*Z* = 4
Mo *K*α radiation
μ = 0.22 mm^−1^
*T* = 296 K
0.28 × 0.08 × 0.04 mm

### Data collection  

Agilent Xcalibur, Eos, Gemini diffractometer
Absorption correction: multi-scan (*CrysAlis PRO*; Agilent, 2014 ▸) *T*_min_ = 0.850, *T*_max_ = 1.000
11300 measured reflections
5503 independent reflections
3958 reflections with *I* > 2σ(*I*)
*R*_int_ = 0.036

### Refinement  

*R*[*F* ^2^ > 2σ(*F* ^2^)] = 0.050
*wR*(*F* ^2^) = 0.135
*S* = 1.02
5503 reflections
222 parameters
H-atom parameters constrained
Δρ_max_ = 0.37 e Å^−3^
Δρ_min_ = −0.32 e Å^−3^

### 

Data collection: *CrysAlis PRO* (Agilent, 2014 ▸); cell refinement: *CrysAlis PRO*; data reduction: *CrysAlis PRO*; program(s) used to solve structure: *SHELXS2014* (Sheldrick, 2008 ▸); program(s) used to refine structure: *SHELXL2014* (Sheldrick, 2015 ▸; molecular graphics: *ORTEP-3 for Windows* (Farrugia, 2012 ▸); software used to prepare material for publication: *PLATON* (Spek, 2009 ▸).

## Supplementary Material

Crystal structure: contains datablock(s) global, I. DOI: 10.1107/S2056989015017454/tk5388sup1.cif

Structure factors: contains datablock(s) I. DOI: 10.1107/S2056989015017454/tk5388Isup2.hkl

Supporting information file. DOI: 10.1107/S2056989015017454/tk5388Isup3.cml

**Fig. 1.**
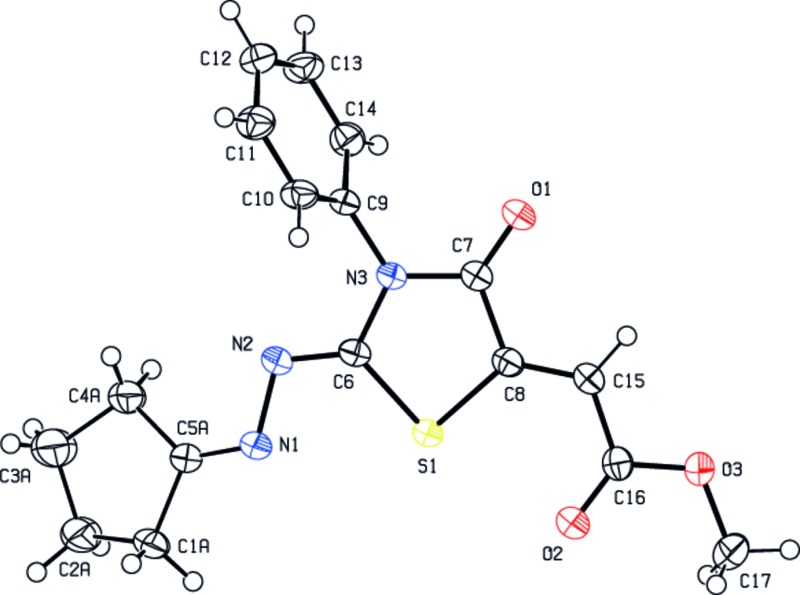
View of the title compound showing only the major component of the disorder. Displacement ellipsoids for non-H atoms are drawn at the 50% probability level.

**Fig. 2.**
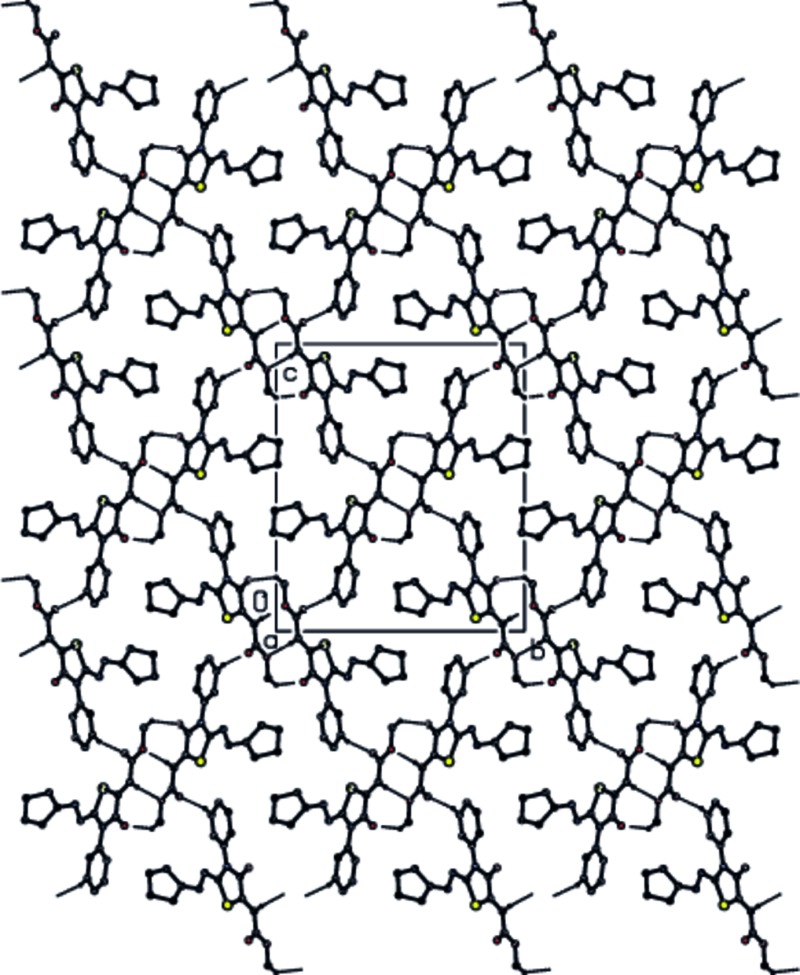
The molecular packing viewed down a axis. The C—H···O interactions are shown as dotted lines with non-participating H atoms omitted for clarity.

. DOI: 10.1107/S2056989015017454/tk5388fig1.tif
View of the title compound showing only the major component of the disorder. Displacement ellipsoids for non-H atoms are drawn at the 50% probability level.

a . DOI: 10.1107/S2056989015017454/tk5388fig2.tif
The mol­ecular packing viewed down *a* axis. The C—H⋯O inter­actions are shown as dotted lines with non-participating H atoms omitted for clarity.

CCDC reference: 1425685

Additional supporting information:  crystallographic information; 3D view; checkCIF report

## Figures and Tables

**Table 1:** Hydrogen-bond geometry (, ) *Cg*4 is the centroid of the C9C14 ring.

*D*H*A*	*D*H	H*A*	*D* *A*	*D*H*A*
C13H13O2^i^	0.93	2.35	3.245(2)	163
C15H15O3^ii^	0.93	2.56	3.485(2)	172
C17H17*A*O1^ii^	0.96	2.42	3.269(2)	147
C3*A*H3*A*2*Cg*4^iii^	0.97	2.96	3.914(3)	169

Symmetry codes: (i) 
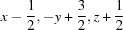
; (ii) 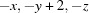; (iii) 
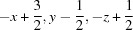
.

## supplementary crystallographic information

### S1. Comment

Thiazoles are important class of heterocyclic compounds, found in many potent biologically active molecules such as sulfathiazol (antimicrobial drug), Ritonavir (antiretroviral drug), Abafungin (antifungal drug), with trade name Abasol cream, and Bleomycine and Tiazofurin (antineoplastic drug). It has been noted over the years that interesting biological activities (Quiroga *et al.*, 2002; Hutchinson *et al.*, 2002) were associated with thiazole derivatives. Applications of thiazoles were found in drug development for the treatment of allergies (Hargrave *et al.*, 1983), hypertension (Patt *et al.*, 1992), inflammation (Sharma *et al.*, 2009), schizophrenia (Jaen *et al.*, 1990), bacteria infection (Tsuji & Ishikawa, 1994), HIV infection (Bell *et al.*, 1995), hypnotics (Ergenc *et al.*, 1999) and for the treatment of pain (Carter *et al.*, 1999), as fibrinogen receptor antagonists with antithrombotic activity (Badorc *et al.*, 1997) and as new inhibitors of bacterial DNA gyrase B (Rudolph *et al.*, 2001). In this context we report in this study the synthesis and crystal structure of the title compound.

The molecular structure of the title compound is shown in Fig. 1. The major and minor components of the disordered cyclo­pentane ring [0.775 (8):0.225 (8)] adopt an envelope conformations with atoms C2A and C2B as the flap in each component. The puckering parameters are Q(2) 0.274 (3) Å, φ (2) = 42.7 (6)° for major component, and Q(2) 0.253 (8) Å, φ (2) = 209.6 (16)° for minor component. The central 1,3-thiazolidine ring (S1/N3C6–C8) makes a dihedral angle of 65.13 (8)° with the phenyl ring (C9–C14).

In the crystal, C—H···O and C—H···π interactions stabilize the molecular packing, forming a three-dimensional network, Fig. 2 and Table 1.

### S2. Experimental

A mixture of 2-cyclopentylidene-*N*-phenylhydrazinecarbothioamide (1 mmol, 233 mg) and dimethyl but-2-ynedioate (1 mmol, 142 mg) in ethylacetate (10 ml) was stirred and refluxed at 350 K. The reaction progress was monitored by TLC until completion. On cooling, a solid yellow product was precipitated, filtered off, dried under vacuum and recrystallized from ethanol to afford yellow crystals.

### S3. Refinement

All H atoms were positioned geometrically and constrained to ride on their parent atoms (C—H = 0.93–0.97 Å) with *U*_iso_(H) = 1.2–1.5*U*_eq_(C). The cyclopentyl group was partially disordered over two positions with refined site-occupancies of 0.775 (8): 0.225 (8) . The (3 0 23), (2 1 24), (-1 3 13), (2 11 22), (5 16 3), (2 2 8), (1 3 27), (4 7 18) and (-1 1 14) reflections were omitted owing to poor agreement.

### Crystal data

**Table:**

C_17_H_17_N_3_O_3_S	*F*(000) = 720
*M**_r_* = 343.40	*D*_x_ = 1.372 Mg m^−^^3^
Monoclinic, *P*2_1_/*n*	Mo *K*α radiation, λ = 0.71073 Å
Hall symbol: -P 2yn	Cell parameters from 2753 reflections
*a* = 5.5215 (3) Å	θ = 4.0–31.8°
*b* = 16.1299 (8) Å	µ = 0.22 mm^−^^1^
*c* = 18.7112 (9) Å	*T* = 296 K
β = 93.980 (5)°	Needle, colourless
*V* = 1662.42 (15) Å^3^	0.28 × 0.08 × 0.04 mm
*Z* = 4	

### Data collection

**Table:**

Agilent Xcalibur, Eos, Gemini diffractometer	5503 independent reflections
Radiation source: Enhance (Mo) X-ray Source	3958 reflections with *I* > 2σ(*I*)
Graphite monochromator	*R*_int_ = 0.036
Detector resolution: 16.0416 pixels mm^-1^	θ_max_ = 32.8°, θ_min_ = 3.3°
ω scans	*h* = −8→6
Absorption correction: multi-scan (*CrysAlis PRO*; Agilent, 2014)	*k* = −21→23
*T*_min_ = 0.850, *T*_max_ = 1.000	*l* = −27→28
11300 measured reflections	

### Refinement

**Table:**

Refinement on *F*^2^	0 restraints
Least-squares matrix: full	Hydrogen site location: inferred from neighbouring sites
*R*[*F*^2^ > 2σ(*F*^2^)] = 0.050	H-atom parameters constrained
*wR*(*F*^2^) = 0.135	*w* = 1/[σ^2^(*F*_o_^2^) + (0.058*P*)^2^ + 0.3068*P*] where *P* = (*F*_o_^2^ + 2*F*_c_^2^)/3
*S* = 1.02	(Δ/σ)_max_ < 0.001
5503 reflections	Δρ_max_ = 0.37 e Å^−^^3^
222 parameters	Δρ_min_ = −0.32 e Å^−^^3^

### Special details

**Table:**

Geometry. Bond distances, angles *etc*. have been calculated using the rounded fractional coordinates. All su's are estimated from the variances of the (full) variance-covariance matrix. The cell e.s.d.'s are taken into account in the estimation of distances, angles and torsion angles
Refinement. Refinement on *F*^2^ for ALL reflections except those flagged by the user for potential systematic errors. Weighted *R*-factors *wR* and all goodnesses of fit *S* are based on *F*^2^, conventional *R*-factors *R* are based on *F*, with *F* set to zero for negative *F*^2^. The observed criterion of *F*^2^ > σ(*F*^2^) is used only for calculating -*R*-factor-obs *etc*. and is not relevant to the choice of reflections for refinement. *R*-factors based on *F*^2^ are statistically about twice as large as those based on *F*, and *R*-factors based on ALL data will be even larger.

### Fractional atomic coordinates and isotropic or equivalent isotropic displacement parameters (Å^2^)

**Table:**

	*x*	*y*	*z*	*U*_iso_*/*U*_eq_	Occ. (<1)
C1A	1.2932 (4)	0.58581 (12)	0.06164 (10)	0.0431 (5)	0.775 (8)
H1A1	1.4493	0.6135	0.0674	0.052*	0.775 (8)
H1A2	1.2277	0.5927	0.0125	0.052*	0.775 (8)
C2A	1.3179 (9)	0.4943 (2)	0.0808 (2)	0.0593 (11)	0.775 (8)
H2A1	1.2026	0.4613	0.0515	0.071*	0.775 (8)
H2A2	1.4806	0.4746	0.0739	0.071*	0.775 (8)
C3A	1.2661 (6)	0.48937 (15)	0.15730 (14)	0.0737 (9)	0.775 (8)
H3A1	1.4154	0.4948	0.1874	0.088*	0.775 (8)
H3A2	1.1932	0.4363	0.1673	0.088*	0.775 (8)
C4A	1.0943 (4)	0.55863 (12)	0.17258 (10)	0.0435 (5)	0.775 (8)
H4A1	0.9287	0.5385	0.1721	0.052*	0.775 (8)
H4A2	1.1381	0.5835	0.2189	0.052*	0.775 (8)
C5A	1.1210 (3)	0.61997 (10)	0.11325 (8)	0.0310 (3)	0.775 (8)
C1B	1.2932 (4)	0.58581 (12)	0.06164 (10)	0.0431 (5)	0.225 (8)
H1B1	1.4095	0.6271	0.0482	0.052*	0.225 (8)
H1B2	1.2079	0.5633	0.0189	0.052*	0.225 (8)
C2B	1.413 (3)	0.5181 (8)	0.1087 (7)	0.0593 (11)	0.225 (8)
H2B1	1.4586	0.4726	0.0786	0.071*	0.225 (8)
H2B2	1.5594	0.5399	0.1333	0.071*	0.225 (8)
C3B	1.2661 (6)	0.48937 (15)	0.15730 (14)	0.0737 (9)	0.225 (8)
H3B1	1.3603	0.4733	0.2008	0.088*	0.225 (8)
H3B2	1.1765	0.4414	0.1386	0.088*	0.225 (8)
C4B	1.0943 (4)	0.55863 (12)	0.17258 (10)	0.0435 (5)	0.225 (8)
H4B1	0.9287	0.5385	0.1721	0.052*	0.225 (8)
H4B2	1.1381	0.5835	0.2189	0.052*	0.225 (8)
C5B	1.1210 (3)	0.61997 (10)	0.11325 (8)	0.0310 (3)	0.225 (8)
C6	0.7150 (3)	0.76671 (10)	0.13570 (8)	0.0265 (3)	
C7	0.3804 (3)	0.85436 (10)	0.14664 (8)	0.0285 (3)	
C8	0.4255 (3)	0.86289 (10)	0.06923 (8)	0.0273 (3)	
C9	0.5666 (3)	0.78876 (9)	0.25645 (8)	0.0252 (3)	
C10	0.7676 (3)	0.81830 (10)	0.29704 (9)	0.0310 (3)	
H10	0.8922	0.8450	0.2752	0.037*	
C11	0.7804 (3)	0.80745 (11)	0.37074 (9)	0.0352 (4)	
H11	0.9157	0.8261	0.3985	0.042*	
C12	0.5927 (3)	0.76895 (11)	0.40315 (9)	0.0352 (4)	
H12	0.5999	0.7628	0.4527	0.042*	
C13	0.3940 (3)	0.73959 (11)	0.36148 (9)	0.0362 (4)	
H13	0.2693	0.7129	0.3833	0.043*	
C14	0.3784 (3)	0.74947 (10)	0.28753 (9)	0.0317 (3)	
H14	0.2443	0.7301	0.2597	0.038*	
C15	0.2862 (3)	0.91153 (10)	0.02523 (9)	0.0299 (3)	
H15	0.1573	0.9405	0.0428	0.036*	
C16	0.3360 (3)	0.91924 (10)	−0.05004 (9)	0.0300 (3)	
C17	0.1970 (4)	0.96748 (13)	−0.16424 (10)	0.0489 (5)	
H17A	0.0682	0.9999	−0.1871	0.073*	
H17B	0.1937	0.9127	−0.1843	0.073*	
H17C	0.3501	0.9931	−0.1716	0.073*	
N1	1.0203 (3)	0.69020 (9)	0.10329 (8)	0.0364 (3)	
N2	0.8698 (3)	0.71279 (9)	0.15868 (7)	0.0318 (3)	
N3	0.5509 (2)	0.80198 (8)	0.18016 (7)	0.0266 (3)	
O1	0.2219 (2)	0.88906 (8)	0.17660 (7)	0.0404 (3)	
O2	0.5119 (2)	0.88964 (8)	−0.07551 (7)	0.0396 (3)	
O3	0.1654 (2)	0.96244 (8)	−0.08814 (6)	0.0387 (3)	
S1	0.67455 (7)	0.80416 (2)	0.04705 (2)	0.02957 (11)	

### Atomic displacement parameters (Å^2^)

**Table:**

	*U*^11^	*U*^22^	*U*^33^	*U*^12^	*U*^13^	*U*^23^
C1A	0.0508 (11)	0.0431 (10)	0.0379 (9)	0.0199 (9)	0.0213 (8)	0.0066 (8)
C2A	0.092 (3)	0.0466 (18)	0.042 (2)	0.0352 (18)	0.0245 (18)	0.0039 (13)
C3A	0.114 (2)	0.0509 (13)	0.0616 (15)	0.0400 (14)	0.0432 (16)	0.0202 (11)
C4A	0.0522 (12)	0.0412 (10)	0.0390 (10)	0.0131 (8)	0.0183 (9)	0.0094 (8)
C5A	0.0314 (8)	0.0372 (8)	0.0249 (7)	0.0078 (6)	0.0061 (6)	0.0017 (6)
C1B	0.0508 (11)	0.0431 (10)	0.0379 (9)	0.0199 (9)	0.0213 (8)	0.0066 (8)
C2B	0.092 (3)	0.0466 (18)	0.042 (2)	0.0352 (18)	0.0245 (18)	0.0039 (13)
C3B	0.114 (2)	0.0509 (13)	0.0616 (15)	0.0400 (14)	0.0432 (16)	0.0202 (11)
C4B	0.0522 (12)	0.0412 (10)	0.0390 (10)	0.0131 (8)	0.0183 (9)	0.0094 (8)
C5B	0.0314 (8)	0.0372 (8)	0.0249 (7)	0.0078 (6)	0.0061 (6)	0.0017 (6)
C6	0.0262 (7)	0.0299 (7)	0.0241 (7)	0.0031 (6)	0.0061 (6)	−0.0022 (6)
C7	0.0275 (7)	0.0308 (8)	0.0277 (8)	0.0043 (6)	0.0055 (6)	−0.0003 (6)
C8	0.0259 (7)	0.0287 (7)	0.0281 (7)	0.0029 (6)	0.0065 (6)	0.0000 (6)
C9	0.0258 (7)	0.0279 (7)	0.0226 (7)	0.0045 (5)	0.0067 (6)	−0.0012 (5)
C10	0.0266 (7)	0.0393 (9)	0.0278 (8)	−0.0046 (6)	0.0069 (6)	−0.0015 (6)
C11	0.0337 (9)	0.0434 (9)	0.0282 (8)	−0.0036 (7)	0.0003 (7)	−0.0051 (7)
C12	0.0416 (9)	0.0398 (9)	0.0249 (8)	0.0028 (7)	0.0073 (7)	0.0027 (7)
C13	0.0353 (9)	0.0414 (9)	0.0332 (9)	−0.0060 (7)	0.0120 (7)	0.0069 (7)
C14	0.0258 (7)	0.0365 (8)	0.0328 (8)	−0.0031 (6)	0.0024 (6)	0.0010 (6)
C15	0.0286 (8)	0.0315 (8)	0.0303 (8)	0.0074 (6)	0.0060 (6)	0.0001 (6)
C16	0.0325 (8)	0.0261 (7)	0.0317 (8)	0.0049 (6)	0.0032 (6)	0.0018 (6)
C17	0.0621 (13)	0.0552 (12)	0.0294 (9)	0.0193 (10)	0.0041 (9)	0.0069 (8)
N1	0.0401 (8)	0.0432 (8)	0.0271 (7)	0.0175 (6)	0.0117 (6)	0.0042 (6)
N2	0.0333 (7)	0.0376 (7)	0.0252 (6)	0.0121 (6)	0.0072 (5)	−0.0004 (5)
N3	0.0254 (6)	0.0325 (7)	0.0224 (6)	0.0064 (5)	0.0052 (5)	−0.0004 (5)
O1	0.0394 (7)	0.0498 (7)	0.0333 (6)	0.0199 (6)	0.0124 (5)	0.0030 (5)
O2	0.0396 (7)	0.0462 (7)	0.0339 (6)	0.0160 (6)	0.0100 (5)	0.0039 (5)
O3	0.0409 (7)	0.0459 (7)	0.0294 (6)	0.0184 (6)	0.0036 (5)	0.0053 (5)
S1	0.0303 (2)	0.0351 (2)	0.02404 (19)	0.01015 (15)	0.00729 (15)	0.00175 (14)

### Geometric parameters (Å, º)

**Table:**

C1A—C5A	1.506 (2)	C8—S1	1.7435 (15)
C1A—C2A	1.523 (4)	C9—C14	1.380 (2)
C1A—H1A1	0.9700	C9—C10	1.385 (2)
C1A—H1A2	0.9700	C9—N3	1.4400 (19)
C2A—C3A	1.480 (4)	C10—C11	1.387 (2)
C2A—H2A1	0.9700	C10—H10	0.9300
C2A—H2A2	0.9700	C11—C12	1.384 (2)
C3A—C4A	1.506 (3)	C11—H11	0.9300
C3A—H3A1	0.9700	C12—C13	1.384 (3)
C3A—H3A2	0.9700	C12—H12	0.9300
C4A—C5A	1.502 (2)	C13—C14	1.389 (2)
C4A—H4A1	0.9700	C13—H13	0.9300
C4A—H4A2	0.9700	C14—H14	0.9300
C5A—N1	1.270 (2)	C15—C16	1.459 (2)
C2B—H2B1	0.9700	C15—H15	0.9300
C2B—H2B2	0.9700	C16—O2	1.2094 (19)
C6—N2	1.273 (2)	C16—O3	1.3371 (19)
C6—N3	1.3930 (18)	C17—O3	1.449 (2)
C6—S1	1.7648 (16)	C17—H17A	0.9600
C7—O1	1.2087 (18)	C17—H17B	0.9600
C7—N3	1.383 (2)	C17—H17C	0.9600
C7—C8	1.493 (2)	N1—N2	1.4203 (18)
C8—C15	1.341 (2)		
			
C5A—C1A—C2A	104.68 (17)	C14—C9—C10	121.67 (14)
C5A—C1A—H1A1	110.8	C14—C9—N3	119.38 (14)
C2A—C1A—H1A1	110.8	C10—C9—N3	118.91 (13)
C5A—C1A—H1A2	110.8	C9—C10—C11	119.03 (15)
C2A—C1A—H1A2	110.8	C9—C10—H10	120.5
H1A1—C1A—H1A2	108.9	C11—C10—H10	120.5
C3A—C2A—C1A	105.0 (2)	C12—C11—C10	120.30 (16)
C3A—C2A—H2A1	110.7	C12—C11—H11	119.9
C1A—C2A—H2A1	110.7	C10—C11—H11	119.9
C3A—C2A—H2A2	110.7	C11—C12—C13	119.66 (15)
C1A—C2A—H2A2	110.7	C11—C12—H12	120.2
H2A1—C2A—H2A2	108.8	C13—C12—H12	120.2
C2A—C3A—C4A	108.1 (2)	C12—C13—C14	120.90 (15)
C2A—C3A—H3A1	110.1	C12—C13—H13	119.6
C4A—C3A—H3A1	110.1	C14—C13—H13	119.6
C2A—C3A—H3A2	110.1	C9—C14—C13	118.43 (15)
C4A—C3A—H3A2	110.1	C9—C14—H14	120.8
H3A1—C3A—H3A2	108.4	C13—C14—H14	120.8
C5A—C4A—C3A	104.65 (15)	C8—C15—C16	120.29 (14)
C5A—C4A—H4A1	110.8	C8—C15—H15	119.9
C3A—C4A—H4A1	110.8	C16—C15—H15	119.9
C5A—C4A—H4A2	110.8	O2—C16—O3	123.44 (15)
C3A—C4A—H4A2	110.8	O2—C16—C15	123.85 (15)
H4A1—C4A—H4A2	108.9	O3—C16—C15	112.71 (14)
N1—C5A—C4A	129.17 (15)	O3—C17—H17A	109.5
N1—C5A—C1A	121.44 (15)	O3—C17—H17B	109.5
C4A—C5A—C1A	109.38 (14)	H17A—C17—H17B	109.5
H2B1—C2B—H2B2	107.8	O3—C17—H17C	109.5
N2—C6—N3	121.76 (14)	H17A—C17—H17C	109.5
N2—C6—S1	126.11 (12)	H17B—C17—H17C	109.5
N3—C6—S1	112.11 (11)	C5A—N1—N2	113.21 (13)
O1—C7—N3	124.37 (15)	C6—N2—N1	110.00 (13)
O1—C7—C8	125.61 (15)	C7—N3—C6	115.42 (13)
N3—C7—C8	110.00 (12)	C7—N3—C9	122.16 (12)
C15—C8—C7	121.50 (14)	C6—N3—C9	122.32 (13)
C15—C8—S1	126.88 (12)	C16—O3—C17	115.13 (13)
C7—C8—S1	111.62 (11)	C8—S1—C6	90.72 (7)
			
C5A—C1A—C2A—C3A	−26.1 (4)	C4A—C5A—N1—N2	2.6 (3)
C1A—C2A—C3A—C4A	29.1 (4)	C1A—C5A—N1—N2	−178.28 (17)
C2A—C3A—C4A—C5A	−20.2 (4)	N3—C6—N2—N1	−177.41 (14)
C3A—C4A—C5A—N1	−177.5 (2)	S1—C6—N2—N1	3.9 (2)
C3A—C4A—C5A—C1A	3.2 (3)	C5A—N1—N2—C6	−158.03 (17)
C2A—C1A—C5A—N1	−165.2 (3)	O1—C7—N3—C6	178.61 (16)
C2A—C1A—C5A—C4A	14.1 (3)	C8—C7—N3—C6	−2.76 (19)
O1—C7—C8—C15	−0.4 (3)	O1—C7—N3—C9	−4.9 (3)
N3—C7—C8—C15	−179.02 (15)	C8—C7—N3—C9	173.70 (13)
O1—C7—C8—S1	178.92 (15)	N2—C6—N3—C7	−174.81 (16)
N3—C7—C8—S1	0.32 (17)	S1—C6—N3—C7	4.01 (17)
C14—C9—C10—C11	0.5 (2)	N2—C6—N3—C9	8.7 (2)
N3—C9—C10—C11	178.44 (14)	S1—C6—N3—C9	−172.45 (11)
C9—C10—C11—C12	−1.1 (3)	C14—C9—N3—C7	66.1 (2)
C10—C11—C12—C13	1.4 (3)	C10—C9—N3—C7	−111.84 (17)
C11—C12—C13—C14	−1.1 (3)	C14—C9—N3—C6	−117.65 (16)
C10—C9—C14—C13	−0.2 (2)	C10—C9—N3—C6	64.4 (2)
N3—C9—C14—C13	−178.09 (15)	O2—C16—O3—C17	3.7 (3)
C12—C13—C14—C9	0.5 (3)	C15—C16—O3—C17	−176.13 (15)
C7—C8—C15—C16	179.45 (15)	C15—C8—S1—C6	−179.15 (16)
S1—C8—C15—C16	0.2 (2)	C7—C8—S1—C6	1.56 (12)
C8—C15—C16—O2	−6.8 (3)	N2—C6—S1—C8	175.66 (16)
C8—C15—C16—O3	173.02 (15)	N3—C6—S1—C8	−3.10 (12)

### Hydrogen-bond geometry (Å, º)

Cg4 is the centroid of the C9–C14 ring.

**Table:**

*D*—H···*A*	*D*—H	H···*A*	*D*···*A*	*D*—H···*A*
C13—H13···O2^i^	0.93	2.35	3.245 (2)	163
C15—H15···O3^ii^	0.93	2.56	3.485 (2)	172
C17—H17*A*···O1^ii^	0.96	2.42	3.269 (2)	147
C3*A*—H3*A*2···*Cg*4^iii^	0.97	2.96	3.914 (3)	169

Symmetry codes: (i) *x*−1/2, −*y*+3/2, *z*+1/2; (ii) −*x*, −*y*+2, −*z*; (iii) −*x*+3/2, *y*−1/2, −*z*+1/2.
