# Supplementary figures and images for: Crystal structure of methyl (2Z)-2-[(2Z)-2-(2-cyclo­pentyl­idenehydrazin-1-yl­idene)-4-oxo-3-phenyl-1,3-thia­zolidin-5-yl­idene]ethano­ate
Source: Acta Crystallogr E Crystallogr Commun. 2015 Sep 26;71(Pt 10):o776–7. doi: 10.1107/S2056989015017454 (PMC4647441; doi:10.1107/S2056989015017454)

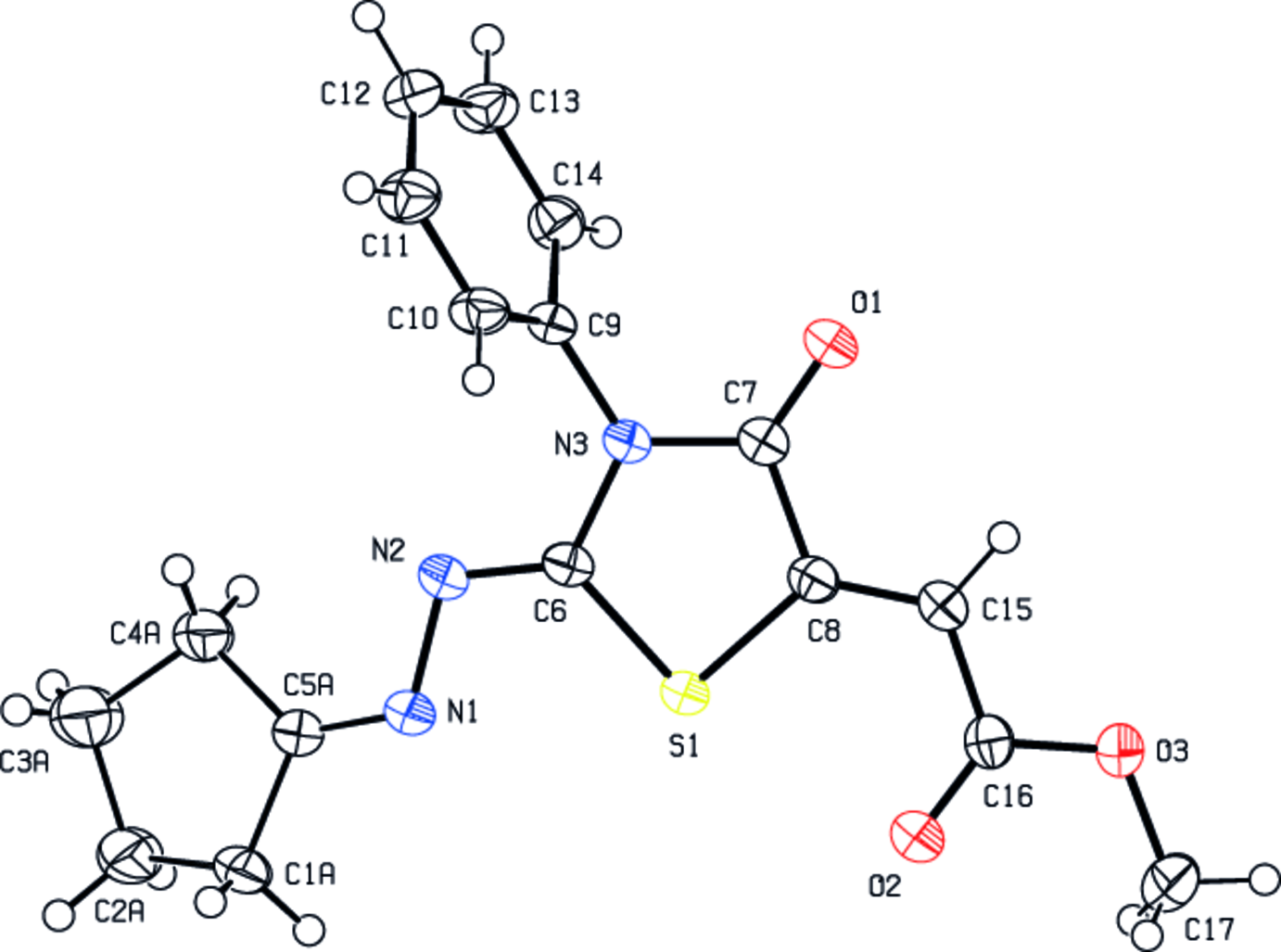

Supplement: Supplementary file 4 [file e-71-0o776-fig1.tif]

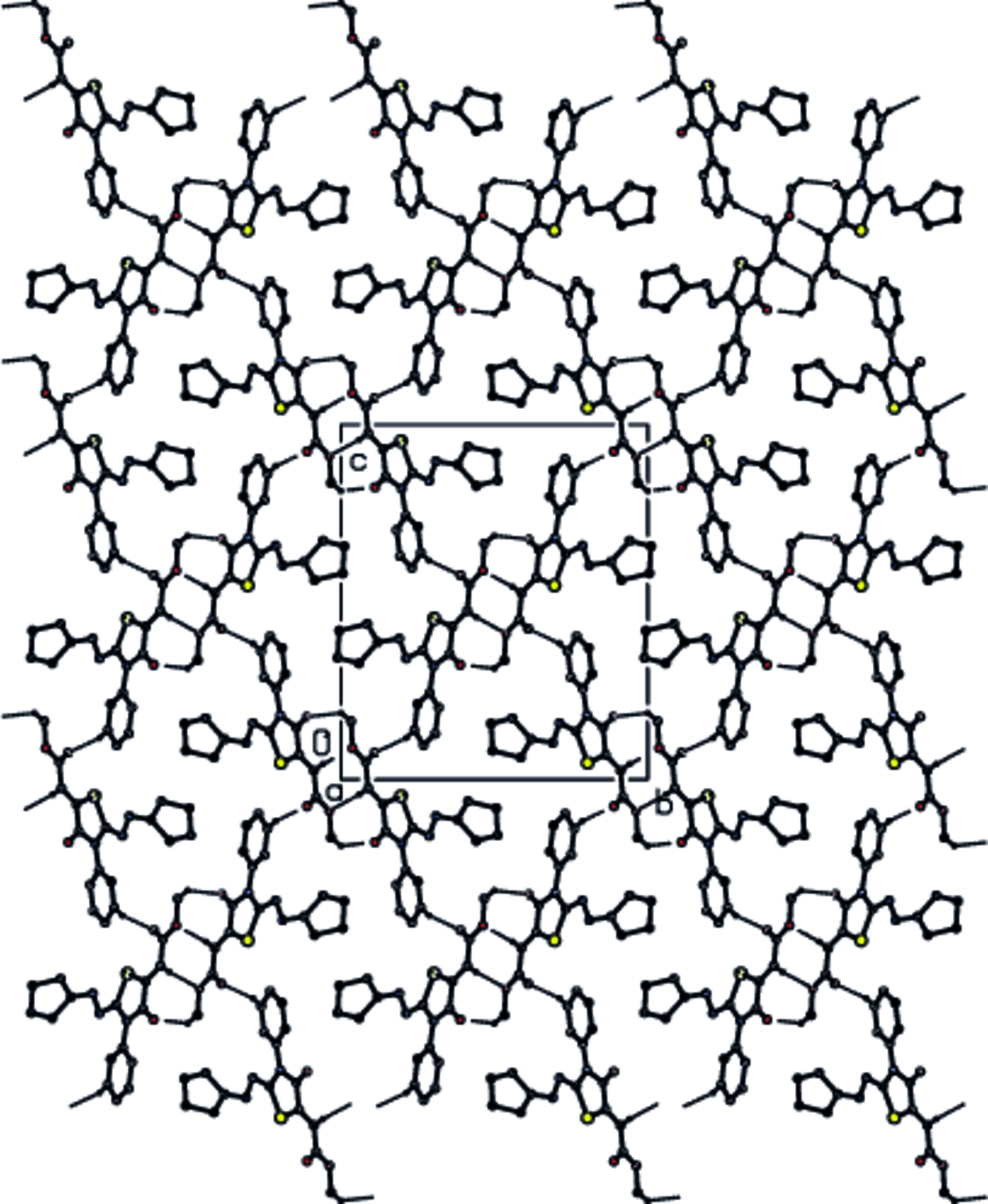

Supplement: Supplementary file 5 [file e-71-0o776-fig2.tif]
